# Non-invasive screening for early Alzheimer’s disease diagnosis by a sensitively immunomagnetic biosensor

**DOI:** 10.1038/srep25155

**Published:** 2016-04-26

**Authors:** Shan-Shan Li, Chih-Wen Lin, Kuo-Chen Wei, Chiung-Yin Huang, Po-Hung Hsu, Hao-Li Liu, Yu-Jen Lu, Sheng-Chi Lin, Hung-Wei Yang, Chen-Chi M. Ma

**Affiliations:** 1 Department of Chemical Engineering, National Tsing Hua University, 101, Section 2, Kuang-Fu Road, Hsinchu 30013, Taiwan, ROC; 2 Department of Neurosurgery, Chang Gung Memorial Hospital, Linkou, 5 Fu-shing Road, Kuei-Shan, Tao-Yuan 33305, Taiwan, ROC; 3 Department of Electrical Engineering, Chang Gung University, 259 Wen-Hwa 1st Road, Kwei-Shan, Tao-Yuan 33302, Taiwan, ROC; 4 Institute of Medical Science and Technology, National Sun Yat-sen University, No.70, Lianhai Road, Gushan District, Kaohsiung 80424, Taiwan, ROC

## Abstract

Amyloid-beta peptide 1–42 (Aβ42) is considered as a reliable biomarker for the early diagnosis of Alzheimer’s disease (AD). Thus, it is urgent to develop a simple and efficient method for the detection of Aβ42. In this work, a reusable biosensor based on magnetic nitrogen-doped graphene (MNG) modified Au electrode for the detection of Aβ42 has been developed. The antibodies of Aβ 1–28 (Aβ_ab_) are used as the specific biorecognition element for Aβ42 that were conjugated on the surface of MNG. In the presence of magnetic nanoparticles on MNG, the electrode coating material, the biosensor can be quickly constructed, without requiring an electrode drying process, which reduce the analysis time and is convenient for proceeding to detection. The reusable biosensor with good reproducibility and stability was linear within the range from 5 pg mL^−1^ to 800 pg mL^−1^, covering the cut-off level of Aβ42 and a detection limit of 5 pg mL^−1^ had been achieved. Furthermore, the fabricated biosensor for Aβ42 detection not only improves the detection performance but also reduces the cost and shortens the response time, demonstrating its potential in diagnosing applications.

Alzheimer’s disease (AD), a progressive neurodegenerative disease affecting a large proportion of the ageing population, is predicted to affect 1 in 85 people globally by 2050^1^. Cognitive function and synaptic integrity of AD patients will gradually lose, neuronal will be selectively dead and abnormal neurotic and core plaques will form in the brains of patients suffering from AD^2^. Since there is no effective cure for the disease to date, once the disease has progressed, the rarely treatment strategies available for AD are useless to patients^3^. Therefore, diagnosing AD at its earliest stages, before obvious symptoms have appeared, is an urgent prerequisite. Current diagnostic imaging techniques for AD, such as positron emission tomography (PET) and magnetic resonance imaging (MRI) imaging which are widely used in hospitals, are insufficient for early diagnosis because they are too expensive for use as conducting regular screening tests. Hence, early diagnosis with body fluids such as cerebrospinal fluid (CSF) is more suitable for early diagnosis^4^. It has been more than 20 years since it was first proposed that deposition of β-amyloid peptides (Aβ) in plaques in brain tissue may cause the neurodegeneration in AD. Among the various Aβ species in human CSF, Aβ42, a peptide of 42 amino acids, is the major constituent of the abnormal plaques in the brains of AD patients. Aβ42 is also considered as a promising biomarker for AD diagnosis, some reports indicate that Aβ42 pathophysiology not only lead to plaque deposition but also can accelerate antecedent limbic and brainstem tauopathy^4^,^5^,^6^. Regarding to the close relationship between AD development and various Aβ_42_ level were reported that most of patients with AD had lower CSF levels of Aβ42^7^,^8^,^9^. Although researches have performed either with the plasma or CSF levels of Aβ42^10^, but some plasma levels of Aβ42 were non-statistically significant between healthy and AD patients^11^,^12^. Thus, CSF levels of Aβ42 is still more suitable representative as biomarker for AD diagnosis^4^.

Up to date, a few methods including enzyme-linked immunosorbent assay (ELISA)^13^, mass spectrometry^14^, surface plasmon resonance (SPR)^15^, scanning tunneling microscopy (STM)^16^, capillary electrophoresis^17^, spectroscopic ellipsometry^18^, gold nanoparticle-based dot-blot immunoassay^19^, metal semiconductor field effect transistor (MESFET)^20^, microchannel electrophoresis^21^ and resonance light scattering^22^ have been developed to detect Aβ species. Nevertheless, most of these methods are usually costly, time-consuming, require complicated instruments or lack sensitivity. Recently, electrochemical biosensors have been widely utilized in food quality control, environmental monitoring and clinical diagnosis due to its simplicity, high sensitivity and rapid response. Some attempts have also been made for the detection of Aβ by electrochemical techniques^23^,^24^.

Graphene, a two-dimensional carbon material, has shown great promise in biomedical applications, including cancer therapy^25^, drug delivery^26^, and biosensors^27^. Besides, graphene based sensors has been mostly used for the detection of glucose, hemoglobin, hydrogen peroxide (H_2_O_2_), ascorbic acid (AA), uric acid (UA), dopamine (DA) and prostate specific antigen (PSA). However, graphene has rarely been applied in AD diagnosis field^28^. Shao Y. Y. *et al*.^29^ overviewed the electrochemical sensors and biosensors based on graphene and summarized its unique physicochemical properties including large surface area, excellent electrical conductivity, rapid electron transfer and rich surface chemistry. Numerous approaches have been proposed to further tailor and develop the physicochemical and electronic properties of graphene, such as chemical functionalization^30^, electrochemical modification^31^, graphene hybrids^32^ and chemical doping with foreign atoms^33^. Among these methods, chemical doping is considered as an effective approach to improve the electrical conductivities^34^. Nitrogen, the atom which has a similar atomic size and contains five valence electrons available to form strong balance bonds with carbon atoms, is consider to be a potential element for the chemical doping of carbon materials. It has been revealed that N doping improved the biocompatibility and sensitivity of carbon nanotubes (CNTs) for the application of biosensing^35^. Consequently, N doping is of great potential to be used for graphene modification. So far, only few studies have been aimed at applying N-doped graphene to electrochemical biosensing. Additionally, a few researchers have deposited Fe_3_O_4_ magnetic nanoparticles onto the surface of graphene sheets to achieve magnetic graphene-based materials^36^,^37^. Owing to the magnetic properties, the magnetic graphene-based materials can be easily coated onto the electrode using magnets and the electrode surface can be regenerated by switching off the magnet. Therefore, magnetic graphene-based materials could be a promising material for the application of electrochemical biosensors.

In this study, a simple, rapid, reusable and non-invasive screening strategy for early Alzheimer’s disease diagnosis using magnetic N-doped graphene (MNG) modified Au electrode was developed. Superparamagnetic magnetite (Fe_3_O_4_) nanoparticles were deposited onto N-doped graphene (NG) to form MNG. The MNG material was successfully labeled with anti-Aβ antibodies through sulfosuccinimidyl-4-(N-maleimidomethyl)cyclohexane-1-carboxylate (sulfo-SMCC) crosslinking method to form magnetic immunocarriers (Aβ_ab_-MNG) (Fig. 1). The magnetic immunocarriers were dropped onto the Au electrode, where they were trapped by placing an external magnet at the underside of the electrode to carry out electrochemical Aβ detection which was directly related to the diagnosis of Alzheimer’s disease (Fig. 2). The fabricated immunomagnetic biosensor showed high sensitivity and selectivity toward Aβ42 detection, which benefits early Alzheimer’s disease diagnosis and provides a useful platform for bioanalytical and biomedical application.

## Results and Discussion

### Characterization of Graphene Oxide (GO), NG and MNG

**Figure 1:**
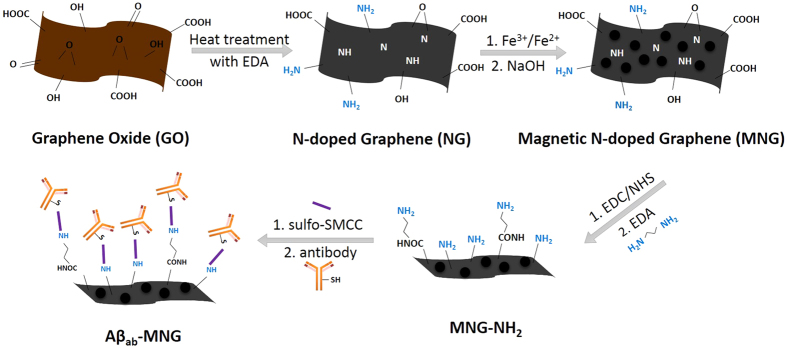
Schematic representation of the preparation of Aβ_ab_-MNG.

**Figure 2:**
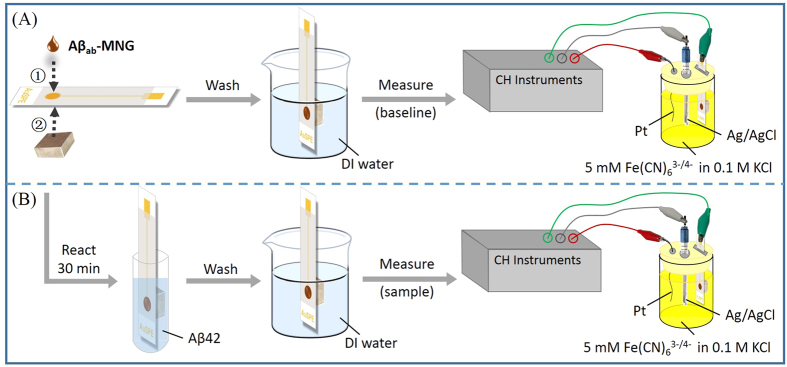
Schematic representation of the electrochemical detection by Aβ_ab_-MNG modified AuSPE (**A**) and the electrochemical detection of Aβ42 using Aβ_ab_-MNG modified AuSPE (**B**).

**Figure 3:**
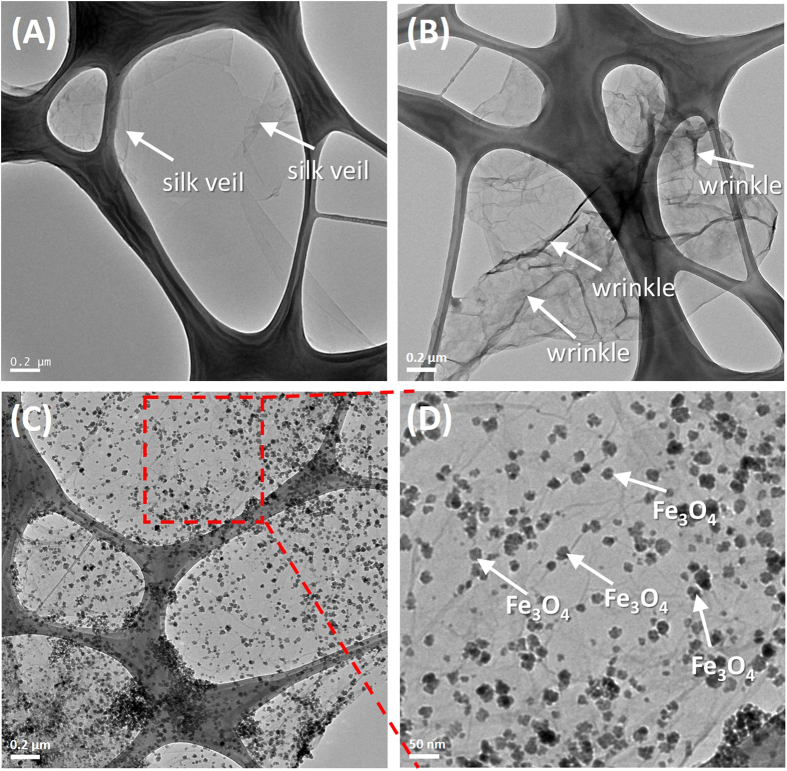
TEM images of GO (**A**), NG (**B**), MNG (**C**) and enlarged image of red area from MNG (**D**).

**Figure 4:**
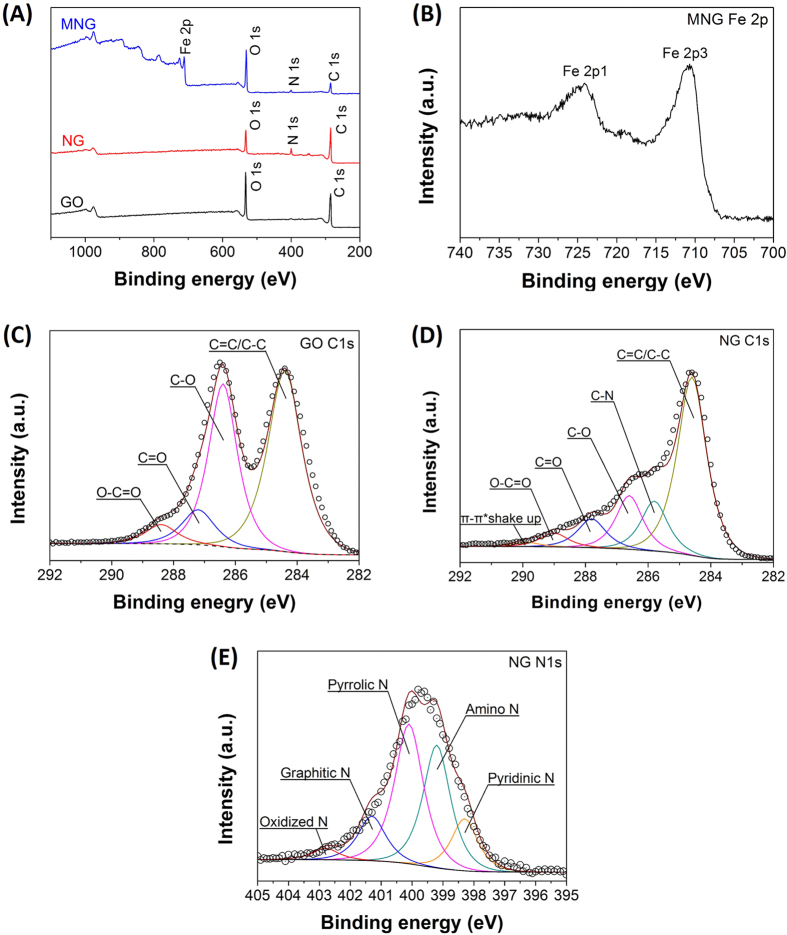
(**A**) XPS survey spectrum of GO, NG and MNG, (**B**) XPS Fe 2p spectra of MNG, (**C**) C1s spectra of GO, (**D**) C1s spectra of NG and (**E**) N1s spectra of NG.

**Figure 5:**
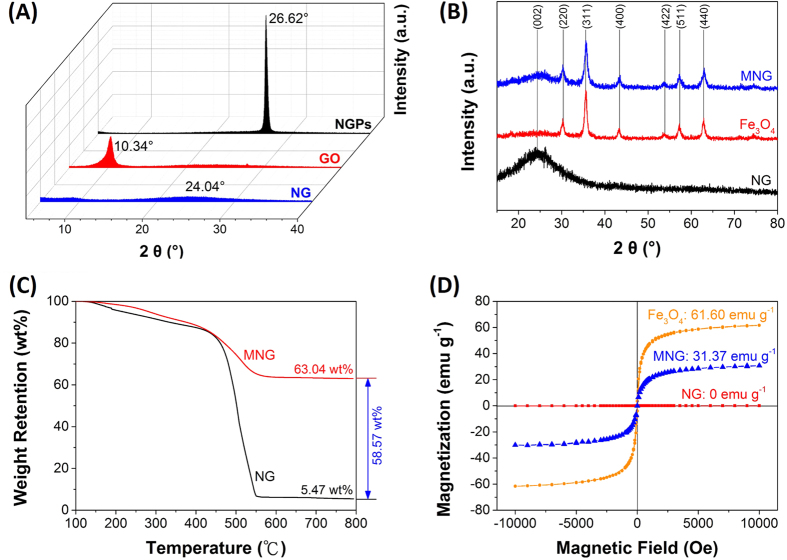
(**A**) X-ray diffraction (XRD) patterns of NGPs, GO and NG. (**B**) XRD patterns of NG, Fe_3_O_4_ and MNG. (**C**) Thermal gravimetric analysis of NG and MNG with a heating rate of 10 °C/min in air. (**D**) Magnetization curves of NG, Fe_3_O_4_ and MNG.

The prepared GO, NG and MNG were characterized by Transmission Electron Microscopy (TEM), as shown in Fig. 1. Different from the silk veil-like structure of GO (Fig. 3A), NG showed a wrinkled, flake-like structure with random stacking (Fig. 3B), which might be attributed to the defective structure formed upon the reduction and the presence of foreign nitrogen atoms^38^. MNG showed that some nanoparticles were attached onto NG sheets (Fig. 3C), as characterized by TEM. Closer examination of MNG revealed that some nanoparticles exhibiting crystal-like morphology with an approximate size of 10~20 nm was attached to the surface of NG sheets (Fig. 3D) which was approved to be Fe_3_O_4_ by X-ray diffraction (XRD) and X-ray photoelectron spectrometer (XPS) investigations. Atomic force microscopy (AFM) images (Fig. S1) showed that the root mean square roughness (Rq) and average roughness (Ra) of MNG was about 3.40 nm and 1.75 nm, which is higher than that of NG (0.280 nm, 0.226 nm), likely due to the attachment of Fe_3_O_4_ to the NG sheets. XPS was used to analyze the surface composition and the chemical configuration of nitrogen atoms in NG. The XPS survey spectra of GO, NG and MNG is shown in Fig. 4A which confirms the existence of N 1s peak in NG and co-existence of N 1s and Fe 2p peaks in MNG composites, indicating the successful nitrogen doping into GO and the formation of Fe_3_O_4_ in MNG composites. The peaks contered at about 285, 400 and 532 eV correspond to the C 1s, N 1s and O 1s, respectively. In the Fe 2p spectrum (Fig. 4B), the peaks at 710.7 and 724.8 eV correspond to Fe 2p_3/2_ and Fe 2p_1/2_^39^ can be observed, which is the indication of the formation of a Fe_3_O_4_ phase in the MNG matrix^40^. Additionally, Fig. 4C,D shows the C 1s XPS spectra of GO and NG. The C 1s of GO can be mainly divided into five peaks, corresponding to C=C/C-C (284.8 ± 0.2 eV), C-O (286.8 ± 0.3 eV), C=O (287.8 ± 0.1 eV), and O-C=O (289.0 ± 0.1eV), respectively^41^. Significantly, the peak intensities of oxygen-containing groups became much weaker in NG while it is worth noting that an additional component appeared at 285.8 eV, which can be attributed to the C-N bonds^41^,^42^. The high resolution N 1s spectrum of NG was shown in Fig. 2E. Generally, the N 1s peaks can be mainly divided into pyridinic- (398.2 eV), pyrrolic- (400.3 eV) and graphitic- (401.4 eV) type of nitrogen atoms doped in the graphene structure^43^,^44^, while the high energy peak at 403 eV is known to be the oxidized nitrogen^45^. Through the preparation process with ethylenediamine, covalent functionalization with amino groups can occur at the edge of defect sites of GO can be generally accepted, thus the peak centered at 399.2 eV can be attributed to animo nitrogen atom^46^. XRD patterns of Nano graphite platelets (NGPs), GO, NG and MNG are shown in Fig. 5A,B. The NGPs diffraction peaks at 2θ = 26.62° were completely replaced by a peak at 10.34°, then the peak was replaced by a broad peak at 20–30°, indicating the oxidation and delamination of NGPs to form GO and the reduction from GO to NG^47^,^48^. Introduction of magnetic particles resulted in XRD-detection of Fe_3_O_4_ peaks within MNG, indicating the successful deposition of Fe_3_O_4_ on NG surface. Thermogravimetric analysis (TGA) with a heating rate of 10 °C/min in air was used to determine the amount of Fe_3_O_4_ in MNG composites. In Fig. 5C, the slight weight loss below 450 °C is attributed to the evaporation of absorbed moisture or gas molecules and the decomposition of labile oxygen functional groups^49^,^50^. A rapid weight loss occures between 450 °C and 550 °C, which can be ascribed to the decomposition of NG sheets in air. Therefore, the weight retention at 800 °C directly translates into the amount of Fe_3_O_4_ in the composites^49^. By using this method, the Fe_3_O_4_ content in MNG was estimated to be about 58.57 wt%. Furthermore, the hysteresis curves were recorded by superconducting quantum interference device (SQUID) (Fig. 5D). The saturation magneticzation of MNG composites was 31.7 emu g^−1^, compared to 0 emu g^−1^ for NG. This value was lower than the magneticization of 61.60 emu g^−1^ for pure Fe_3_O_4_ due to the proportional decrease in Fe_3_O_4_ per unit weight^36^. The magnetization of MNG was not only sufficient to avoid escape from the submerged electrode, but also allowed the rapid construction of the sensor for electrochemical sensing in a magnetic field.

### Optimization of detection conditions

**Figure 6:**
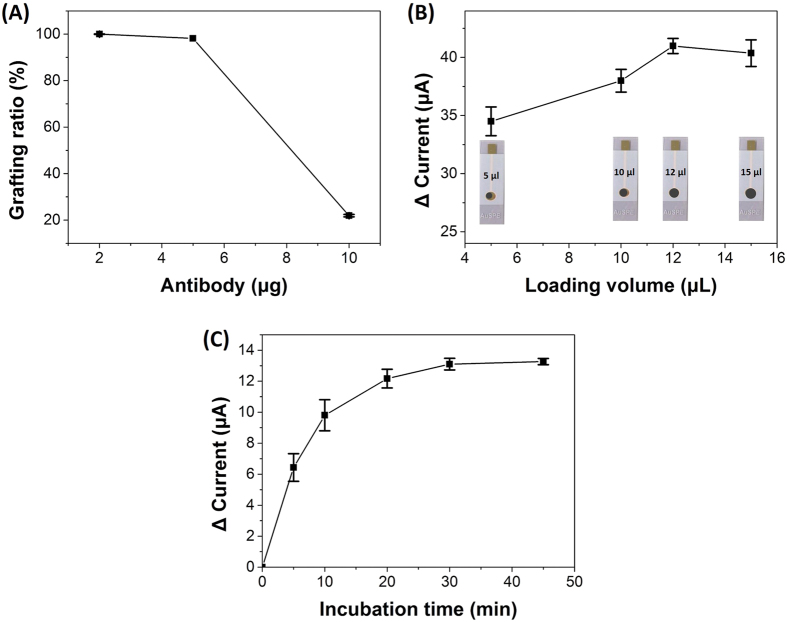
(**A**) The grafting ratio of different weights of Aβ_ab_ reacted with 1 mg MNG. (**B**) The change in current by different volumes of Aβ_ab_-MNG dropped onto Au electrode. (**C**) The change in current by various incubation times of Aβ_ab_-MNG modified Au electrode with 800 pg mL^−1^ Aβ42. Error bars represent the standard deviation (SD) from three independent determinations.

The amount of Aβ_ab_ immobilized onto MNG would affect the detection range of Aβ42, because the more Aβ_ab_ immobilized onto MNG that could capture more Aβ42 peptide. Enzyme-linked immunosorbent assay (ELISA) was used to determine the loading efficiency of Aβ_ab_ onto MNG at a wavelength of 492 nm, which was chosen based on the absorption spectrum of unbound fluorescein isothiocyanate-labeled Aβ_ab_ (FITC-Aβ_ab_)^51^. The supernatants were measured after reacting MNG with various weights of Aβ_ab_. The grafting ratio decreased with the increased weight of Aβ_ab_, because the limited amine (NH_2_) groups on MNG were not enough to conjugate more Aβ_ab_. The grafting ratio was 100% when the weight of Aβ_ab_ we added to conjugate with 1 mg MNG was 2 μg. If the weight of Aβ_ab_ we added increased to 5 μg while the weight of MNG remained 1 mg, the grafting ratio decreased to 98%. In addition, if 1 mg of MNG immobilized with 2 μg Aβ_ab_ was utilized to detect Aβ42 concentration, a wide detection range which covered the cut-off level of Aβ42 will be obtained. Owing to the cost concern, the optimal amount of antibodies immobilized on MNG was chosen to be 2 μg for 1 mg of MNG (Fig. 6A). We further investigated the effect of the loading volume of Aβ_ab_-MNG drop-deposited on the Au electrode. With an increasing volume loaded onto Au electrode, the change of the current increased. The optimum volume was found to be 12 μL, fully covering the sensing area of the Au electrode and possessing a stable current response (Fig. 6B).

The incubation time of the electrode with Aβ42 (800 pg mL^−1^) is also the important parameter that would affect the analytical performance. The result showed the current increased with increasing the incubation time, but the current would trend to a constant value after 30 min of incubation time (Fig. 6C). Thus, in order to reduce the time for total Aβ42 immobilization and maintain the activity of Aβ42, the incubation time of 30 min was selected in this study.

In summary, an Aβ_ab_-MNG-modified Au electrode was rapidly constructed by the deposition of 12 μL of Aβ_ab_-MNG (2 μg Aβ_ab_ per 1 mg MNG) aqueous dispersion on an Au electrode surface under a magnetic field. In other words, a biosensor was formed without requiring a drying step, which saves the time significantly, and the sensor was then incubated with 1 mL of Aβ42 for 30 min. The entire procedure was faster and more convenient than other methods, such as ELISA. The response time of this study (30 min) was reduced 9 to 10-fold compared with ELISA method (typically requires at least 4.5–5 h^52^,^53^).

### Electrochemical characterization of the Aβ_ab_-MNG-modified Au electrode

**Figure 7:**
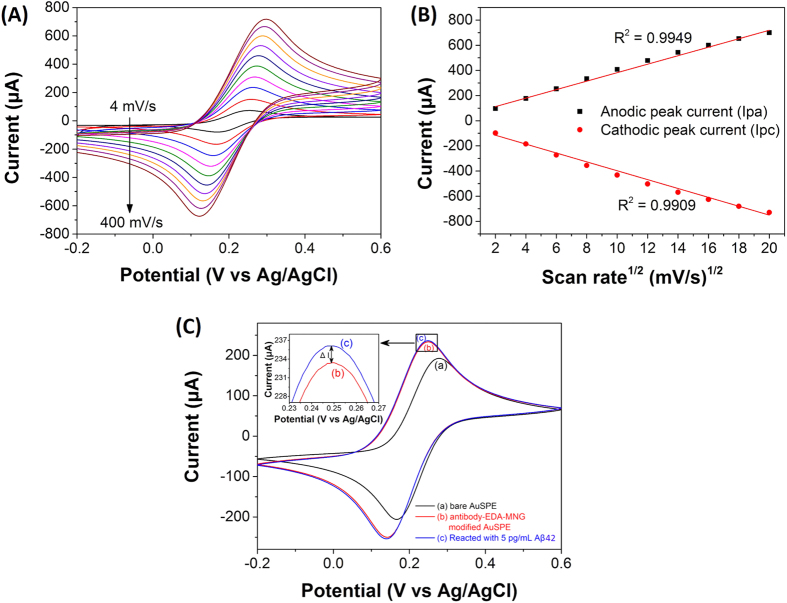
(**A**) Cyclic voltammograms of Aβ_ab_-MNG modified AuSPE in 0.1 M KCl with 5 mM Fe(CN)_6_^3−^/Fe(CN)_6_^4−^ at different scan rates (from black to brown): 4, 16, 36, 64, 100, 144, 196, 256, 324, and 400 mV s^−1^. (**B**) The plot of anodic (Ipa) and cathodic (Ipc) peak current versus square root of the scan rate (*v*^1/2^). (**C**) Cyclic voltammograms bare AuSPE (black), Aβ_ab_-MNG modified AuSPE (blue) and Aβ_ab_-MNG modified AuSPE reacted with 5 pg mL^−1^ of Aβ42 (red).

The electrochemical behavior of the Aβ_ab_-MNG-modified Au electrode was studied by cyclic voltammetry (CV) and differential pulse voltammetry (DPV) in 0.1 M KCl solution with 5 mM K_3_[Fe(CN)_6_]/K_4_[Fe(CN)_6_]. The pH was maintained at 7.0 because the pH of blood samples was usually neutral. All measurements were conducted at room temperature. In this study, K_3_[Fe(CN)_6_]/K_4_[Fe(CN)_6_] was used as electron transfer mediator, providing a convenient and valuable approach for analyzing the electron transfer between the solution and the electrode surface. The influence of CV scan rate on the electrochemical behavior of Fe(CN)_6_^3−/4−^ on Aβ_ab_-MNG-modified Au electrode was investigated and the results are shown in Fig. 7A. The peak of the anodic and the cathodic currents increased linearly with the square root of scan rate (*v*^1/2^) over the range of 4 to 400 mV/s (Fig. 7B), indicating that the redox reaction between Fe(CN)_6_^3−/4−^ and Aβ_ab_-MNG-modified Au electrode is a diffusion-controlled process^54^. In Fig. 7C, the results showed that the current of Aβ_ab_-MNG-modified Au electrode was higher than that of bare Au electrode, and the current was further slightly increased after adding 5 pg mL^−1^ of Aβ42, indicating that the Aβ_ab_-MNG was indeed deposited on the Au electrode and the electrode can capture the Aβ42 in the solution.

### Analytical performance

**Figure 8:**
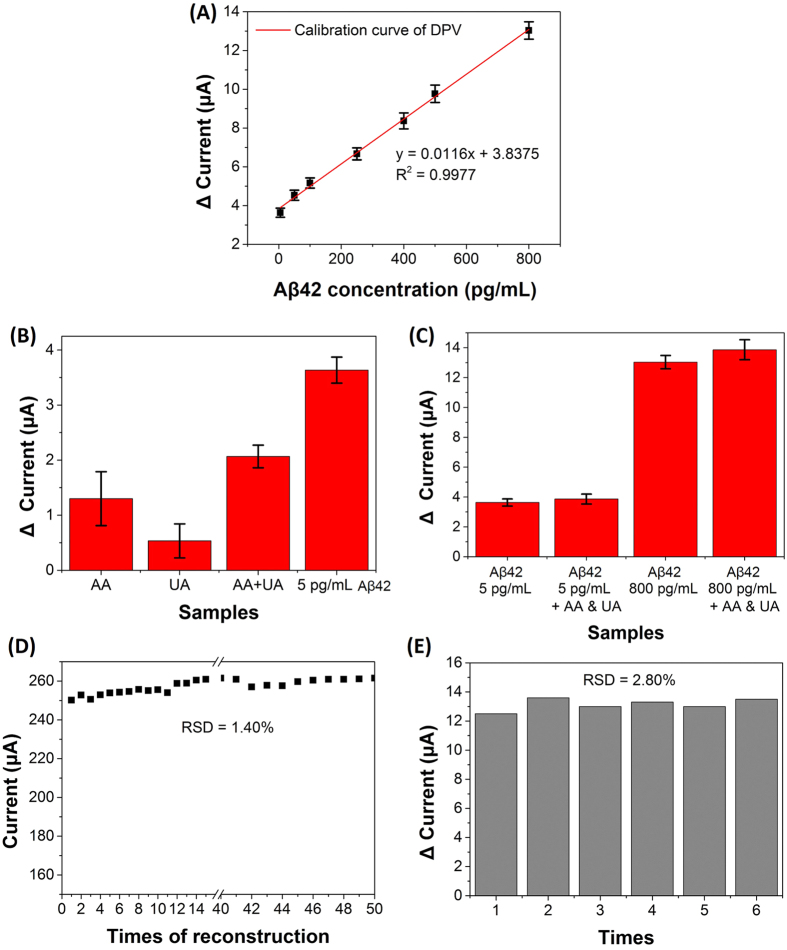
(**A**) Calibration curve for detection of Aβ42 from 5 to 800 pg mL^−1^. (**B**) Change in current of various interference species. (**C**) Change in current of Aβ42 detection with or without interference species. Error bars represent the standard deviation (SD) from three independent determinations. (**D**) Reusability of the Aβ_ab_-MNG modified AuSPE biosensor. (**E**) The change in current for the detection of 800 pg mL^−1^ Aβ42.

Under optimum conditions, the current change (ΔC) after reacted with various concentrations (5, 50, 100, 250, 400, 500 and 800 pg mL^−1^) of Aβ42 was obtained from differential pulse voltammetry (DPV) using the fabricated electrochemical biosensor. The ΔC increased with increasing concentration of Aβ42 in the incubation solution (Fig. 8A). The calibration curve showed a good linear relationship between the ΔC and the Aβ42 concentration in the range from 5 pg mL^−1^ to 800 pg mL^−1^ with a correlation coefficient of 0.9977, indicating that the response was the direct result of Aβ42 binding to the Aβ_ab_-MNG through antigen-antibody recognition. This wide detection range covered these cut-off CSF levels of Aβ42 (603, 192, 500, 457 pg mL^−1^)^7^,^8^,^9^,^12^, illustrating that the biosensor can be utilized for the diagnosis of AD. These differences cut-off levels in observations might be due to the variations in sample assaying protocols and selection of patient groups. The limit of detection was 5 pg mL^−1^ which was much lower than those reported previously^23^,^55^,^56^.

To investigate the selectivity of the biosensor, typical interfering species were incubated with the Aβ_ab_-MNG modified Au electrode. According to the levels in human cerebrospinal fluid (CSF), the following interfering species were used: ascorbic acid (AA, 129 μM) and uric acid (UA, 17.7 μM)^57^. The current changes of AA, UA or mixture of AA and UA were much lower than that of 5 pg mL^−1^ Aβ42 (Fig. 8B). Besides, the changes in current after the incubation of Aβ42 in the presence of the interfering species (3.87 ± 0.33 μA for 5 pg mL^−1^, 13.87 ± 0.66 μA for 800 pg mL^−1^) were not significantly different compared to the treatment with 5 pg mL^−1^ or 800 pg mL^−1^ Aβ42 (3.63 ± 0.24 μA for 5 pg mL^−1^, 13.03 ± 0.45 μA for 800 pg mL^−1^) alone (Fig. 8C). These results indicated that the Aβ_ab_-MNG modified immunosensor biosensor resisted interference well.

### Reusability, reproducibility and precision

The fabricated immunosensor can be quickly reconstructed because of the superparamagnetic property of Aβ_ab_-MNG. Thus, we further investigated the reusability data of bare screen-printed Au electrode (AuSPE) that was reconstructed with Aβ_ab_-MNG. After reconstructing the sensor for 50 times, the current response remained in a range of 2.5~2.6 × 10^−4^ A with a relative standard deviations (RSD) of 1.4% (Fig. 8D), confirming the good reusability. The Au electrode could be repeatedly used at least 50 times. Furthermore, we also investigated the precision of the fabricated immunosensor. AuSPE was repeated to reconstruct by Aβ_ab_-MNG and reacted with 800 pg mL^−1^ Aβ42 of each (n = 6). The RSD was 2.8% in six times, showing good precision and acceptable fabrication reproducibility (Fig. 8E). These results indicated that the immunosensor had acceptable reliability and stability.

## Conclusion

We first reported an electrochemical strategy for the sensitive detection of Aβ42 using graphene based biosensor^28^. The obtained MNG was characterized by various techniques confirming that the nanoscale magnetic nanoparticles was homogeneous distributed on the nitrogen-doped graphene sheet. Owing to the magnetic property of MNG, the Aβ_ab_-MNG solution can be drop-coated onto the surface of Au electrode by placing an external magnet at the underside of the electrode to rapidly construct a biosensor for the detection of Aβ42, and the biosensor can be easily and conveniently regenerated by switching off the magnetic field used to capture the magnetic materials onto the electrode surface. The fabricated biosensor showed good stability and reusability (RSD = 1.40%, n = 50), yielding a limit of detection of 5 pg mL^−1^. The simplicity, reusability, reproducibility, stability, high sensitivity and selectivity, low cost, as well as quick response time of the method facilitated the measurements of the concentration of Aβ42. It is believed that this work would be valuable in the early diagnosis of AD and lead to many applications in the design of sensitive electrochemical biosensors.

## Methods

### Chemicals and instrumentation

Nano graphite platelets (NGPs) was obtained from Angstron Materials LLC, Dayton, OH, USA. Sulfuric acid (H_2_SO_4_) (97%), sodium nitrate (NaNO_3_), potassium permanganate (KMnO_4_), hydrogen peroxide (H_2_O_2_) (35%), iron(III) chloride hexahydrate (FeCl_3_·6H_2_O), potassium chloride (KCl), potassium ferricyanide (K_3_[Fe(CN)_6_]) and potassium hexacyanoferrate(II) trihydrate (K_4_[Fe(CN)_6_]·3H_2_O) were received from Showa Chemical Co., Ltd., Tokyo, Japan. Hydrochloric acid (HCl) was purchased from Union Chemical Work Ltd., Hsinchu, Taiwan. Sodium hydroxide (NaOH) was obtained from Sigma Co., Tokyo, Japan. 2-(N-morpholino)ethanesulfonic acid hydrate (MES hydrate) and bovine serum albumin (BSA) were received from Sigma Co., St. Louis, MO, USA. Iron(II) chloride tetrahydrate (FeCl_2_·4H_2_O) and ethylenediamine (EDA) were purchased from Acros Organics, Morris Plains, NJ, USA. Sulfo-N-hydroxysuccinimide (Sulfo-NHS), asobic acid (AA) and uric acid (UA) were purchased from Sigma-Aldrich Co., LLC, Tokyo, Japan. Sulfosuccinimidyl-4-(N-maleimidomethyl)cyclohexane-1-carboxylate (sulfo-SMCC) was obtained from Thermo Fisher Scientific Inc., Waltham, MA, USA. 1-(3-Dimethylaminopropyl)-3-ethylcarbodiimide (EDC) was purchased from Alfa Aesar, Heysham, Lancashire, UK. Beta-amyloid [1–28] antibody and beta-amyloid [1–42] peptide were provided by Abbiotec, LLC. San Diego, CA, USA. Deionized (DI) water was used through out the experiment.

The surface morphologies of materials were studied by a transmission electron microscope (TEM, JEM-2100, JEOL), and scanning probe microscope system (SPM, Dimension ICON, Bruker). The spectrum analysis of materials were studied by X-ray photoelectron spectroscopy analysis (XPS, PHI Quantera SXM using an Al Ka X-ray source, ULVAC-PHI) and X-ray diffraction spectroscopy (XRD, ID3000, SCINTAG). The magnetic and thermal property of materials were studied by superconducting quantum interference device (SQUID, Quantum Design SQUID magnetometer MPMS-5, Quantum Design) and thermogravimetric analysis (TGA, SDT Q600, TA Instruments), respectively. The result of ELISA was performanced by Synergy HT Multi-Mode Microplate Reader (Synergy™ HT, BioTek). All of electrochemical analysis was performanced by electrochemical equipment (CHI628D, CH Instruments) used a standard three-electrode cell. Au electrode as the working electrode was used a bare screen-printed Au electrode (AuSPE) was obtained from Zensor R&D, Taichung, Taiwan, an Ag/AgCl electrode (3 M KCl, 0.207 V vs. SHE at 25 °C) and a platinum wire were employed as the reference and counter electrode, respectively.

### Synthesis of GO

GO was prepared from NGPs powders by modified Hummers’ method^58^. 0.25 g NGPs, 0.125 g NaNO_3_ and 12 mL 98% H_2_SO_4_ were well mixed in a flask in ice bath, then 0.75 g KMnO_4_ was added slowly and ultrasonicated for 2 hours, keeping the temperature below 5 °C in this step. 12 mL deionized water (DI water) was added to the mixture slowly and maintain 90 °C for half an hour, followed by the addition of 50 mL 10% H_2_O_2_ to terminate the reaction. For the purification of GO, the solution was centrifuged at 10,000 rpm followed by washing with DI water several times until pH reached neutral. Finally, the subnatant was further purified by dialysis for one week to remove the remaining metal species to obtain GO suspension.

### Preparation of NG and MNG

The GO suspension was diluted to 1 mg mL^−1^, and then 120 mL of the solution and 3 mL EDA were mixed in a 250 mL flask. The mixture was reacted for 48 hours at 60~65 °C with a magnetic stirring. After the reaction, the mixture was filtered, washed with DI water to obtain nitrogen-doped graphene (NG) (Fig. 7).

Magnetic nitrogen-doped graphene (MNG) was synthesized by coprecipitation of FeCl_3_ and FeCl_2_·4H_2_O in the presence of NG (Fig. 7). Briefly, 200 mg of NG in 20 mL of DI water was ultrasonicated for 30 min. The mixture of FeCl_3_·6H_2_O (4.32 mmol) and FeCl_2_·4H_2_O (6.48 mmol) dissolved in 380 mL DI water at room temperature was added to the NG suspension and stirred for 5 min under N_2_ gas. The solution was heated slowly to 60 °C and 30 mL of 0.576N NaOH was added over a 10 min period. When NaOH was all added, the temperature was around 80 °C. The solution was then rapidly quenched in an ice bath to terminate the reaction. The magnetic material (MNG) can be separated from the solution by attraction to the wall of a separation funnel using a strong magnet. MNG was washed several times with DI water to remove the unreacted material, and then uniformly dispersed in DI water by sonication at 300 W for 1 h.

### Preparation of amine-terminated MNG (MNG-NH_2_) and MNG-Aβ_ab_

MNG was modified with ethylenediamine (EDA) to form amine-terminated MNG (MNG-NH_2_) (Fig. 7). Briefly, 540 mg of sulfo-NHS and 480 mg of EDC·HCl were dissolved in 20 mL of 0.5 M MES buffer (pH = 6.3) away from light. A 40 mL aliquot mixed with 20 mL of MNG (10 mg mL^−1^) at 25 °C and reacted for 30 min in dark place to allow the formation activated carboxyl groups of MNG. Activated MNG was separated, washed with 0.1 M MES buffer, resuspended in 20 mL of DI water, and then mixed with 5 mL of EDA at 25 °C by vortexing for 1 h followed by washing with DI water.

The thiol group of the fragment crystallizable region (F_c_) of Aβ_ab_ would be specifically conjugated onto MNG-NH_2_ via sulfo-SMCC crosslinker, so the antigen-binding fragent (F_ab_) may be outwardly exposed enhancing the binding affinity between the antigen and antibody. 0.05 mL of MNG-NH_2_ (10 mg mL^−1^) was mixed with 0.05 mL of sulfo-SMCC (5 mg mL^−1^) at 25 °C and reacted for 60 min by vortexing. The material was separated, washed with DI water, resuspended in 500 μL DI water, and then mixed with 10 μL of Aβ_ab_ at 25 °C by vortexing for 2 h (Fig. 7). The Aβ_ab_-MNG was then separated from the solution, washed with DI water to remove the unbound Aβ_ab_, and dispersed in 500 μL of DI water. In the last step, Aβ_ab_-MNG was blocked with 2% BSA solution for 1 h.

### Fabrication of Aβ_ab_-MNG- modified Au electrode

As shown in Fig. 8A, 12 μL of Aβ_ab_-MNG solution (10 mg mL^−1^ in DI water) were drop-deposited onto the surface of an Au electrode (diameter 5 mm; geometric area 0,196 cm^2^) in a magnetic field. Electrochemical measurements were performed with a CHI628D electrochemical workstation (CH Instruments, Austin, TX, USA) at room temperature in 0.1 M KCl solution containing 5 mM K_3_[Fe(CN)_6_] and 5 mM K_4_[Fe(CN)_6_]. A three-electrode system with Aβ_ab_-MNG- modified Au electrode as the working electrode, bare Pt wire as the counter electrode and Ag/AgCl electrode as the reference electrode was used. Differential pulse voltammetry (DPV) measurements were performed over a range of −0.2 V to 0.6 V with a potential step of 0.005 V and pulse amplitude of 0.05 V.

### Aβ42 detection by the Aβ_ab_-MNG- modified Au electrode

Figure 8A shows the response current of the Aβ_ab_-MNG- modified Au electrode in 5 mM (K_3_[Fe(CN)_6_])/(K_4_[Fe(CN)_6_]) and 0.1 M KCl solution was used to establish the baseline current before any samples were measured. For the Aβ42 standard curve, MNG-Aβab-modified Au electrode was soaked in 1 mL of Aβ42 solution with various concentrations for 30 min (Fig. 8B).

## Additional Information

**How to cite this article**: Li, S.-S. *et al*. Non-invasive screening for early Alzheimer’s disease diagnosis by a sensitively immunomagnetic biosensor. *Sci. Rep.* **6**, 25155; doi: 10.1038/srep25155 (2016).

## Supplementary Material

Supplementary Information
