# Supplementary material for: Non-invasive screening for early Alzheimer’s disease diagnosis by a sensitively immunomagnetic biosensor
Source: Sci Rep. 2016 Apr 26;6:25155. doi: 10.1038/srep25155 (PMC4844990; doi:10.1038/srep25155)
Supplement: Supplementary Information [file srep25155-s1.doc]

**Supplementary Figure**

Non-invasive screening for early Alzheimer’s disease diagnosis by a sensitively immunomagnetic biosensor

Shan-Shan Li^a, 1^, Chih-Wen Lin^a, 1^, Kuo-Chen Wei^b, 1^, Chiung-Yin Huang^b^, Po-Hung Hsu^c^, Hao-Li Liu^c^, Yu-Jen Lu^b^, Sheng-Chi Lin^a^, Hung-Wei Yang^d,*^, Chen-Chi M. Ma^a,*^

^a^ Department of Chemical Engineering, National Tsing Hua University, 101, Section 2, Kuang-Fu Road, Hsinchu 30013, Taiwan, ROC

^b^ Department of Neurosurgery, Chang Gung Memorial Hospital, Linkou, 5 Fu-shing Road, Kuei-Shan, Tao-Yuan 33305, Taiwan, ROC

^c^ Department of Electrical Engineering Chang Gung University Kuei-Shan, Tao-Yuan 33302, Taiwan, ROC

^d^ Institute of Medical Science and Technology, National Sun Yat-sen University, Kaohsiung 80424, Taiwan, ROC


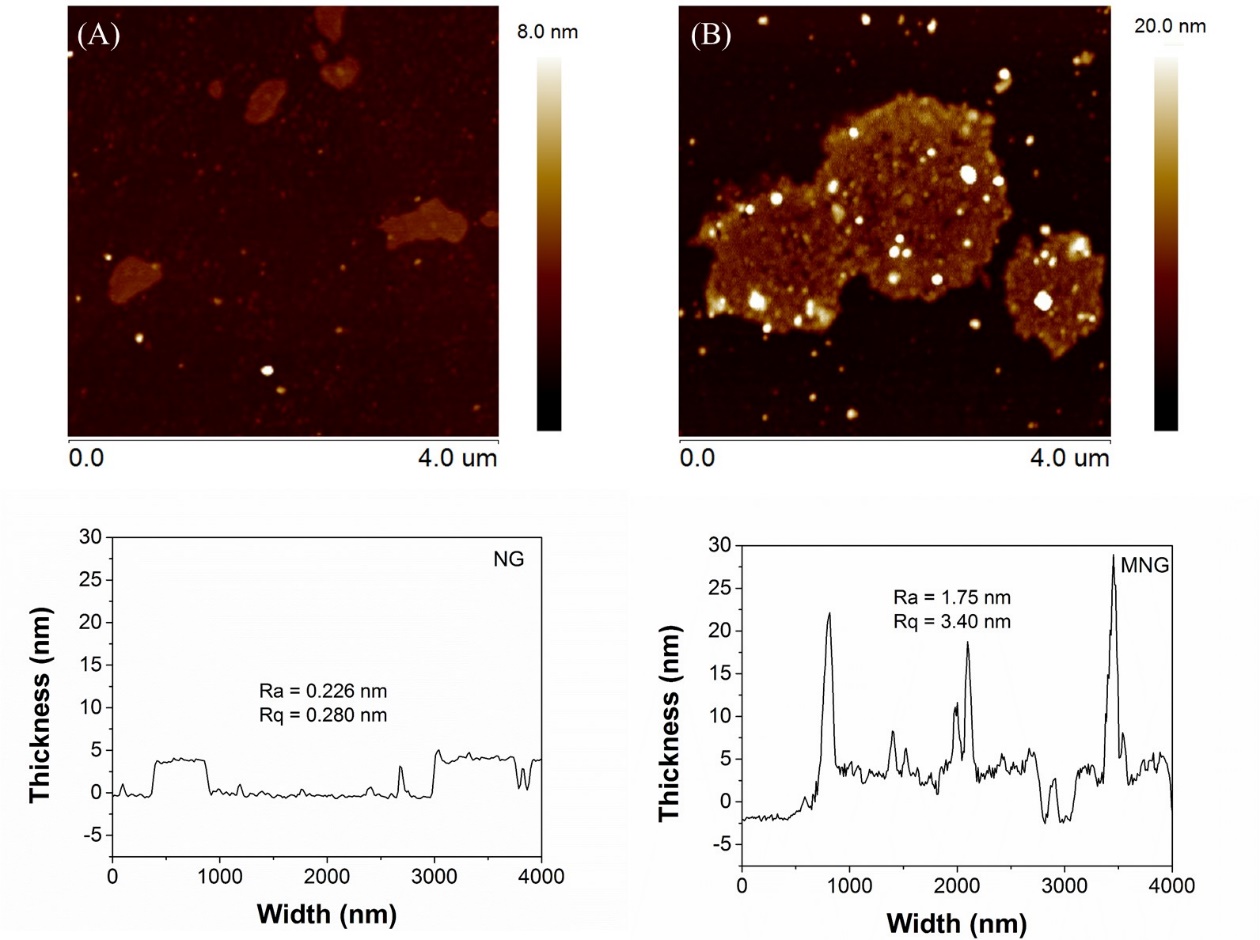


**Figure S1.** AFM images of NG (A) and MNG (B) (top: 2D photographs; bottom: curves of thickness distribution).
